# On the Treatment of Fever by Large Doses of Sulphate of Quinia at St. George’s Hospital

**Published:** 1853-10

**Authors:** A. White Barclay


*On the Treatment of Fever by large doses of Sulphate of Quinia at St. George's Hospital.* By A. White Barclay, M. D.; and Dr. Dundas.—The cases treated by quinia, writes Dr. Barclay, may be divided into three classes.—1. Those in which its exhibition was followed by marked depression. 2. Those in which the pulse became slower, without general prostration or sickness. 3. Those in which no decided effect was produced which could be noted at the time.

1. Including all the cases together in which this effect was produced, the number is five. Two have been already mentioned as fatal, one of apparent typhus, complicated with albuminuria, which was not detected until the subsidence of the fever, and ultimately proving fatal. The physiological effect of the remedy was produced by very different quantities in different instances, and given at very varying intervals. One patient took twenty grains every three hours for nine times; another took ten grains every two hours for ten times: while a third took twenty grains every six hours for only three times; the other two had twenty grains every four and every six hours respectively for eight times.

Of the 3 uncomplicated cases, 1 died, 1 was ill ten days before admission, and remained under treatment forty five days before recovery was complete; the other had been ill a week, and was discharged cured at the end of twenty-four days.

2. In 2 instances only did the pulse become remarkably slower without any depression: one took ten grains every three hours, the other fifteen grains every four hours for about two days, after which the dose was gradually diminished. The first had been ill five days, and was discharged cured in eleven days, having been kept under observation longer than was perhaps absolutely needed to ascertain that recovery was really complete; the second had been ill only two days, and got well in three weeks.

The first case was not severe, and had no spots; the second was delirious for the first two or three nights, and had a faint rather indistinct rash on the abdomen; he had also pretty severe diarrhœa, but no evidence of ulceration of the bowels. The pulse fell in each below 50, but it is necessary to state here, that in another instance it fell still lower in which no quinia was given. He made a very rapid recovery, being ill only five days before admission, and leaving the hospital cured in eight days.

3. In 11 cases there was no distinct physiological effect produced by the quinia; and it remains to inquire whether recovery was more rapid under this mode of treatment than any other; and this may be best accomplished by instituting a comparison betwen them and the whole of the other fever patients admitted during the same time. Twelve examples of a very mild form, which might be justly called febricula, are omited, and there then remain 51 instances of well marked fever which were not treated by quinia.

Among these, 20 exhibitited fever spots on the chest or abdomen, and 6 with and 5 without spots unequivocal evidence of ulceration of the bowels. By this is not meant merely the occurences of thin watery motions, which have been observed in the majority of the patients, but the persistence of diarrhœa, with a patchy, shining, or fissured tongue. We have, therefore, as the basis of our analysis, 26 cases which neither had fever spots nor distinct evidence of ulceration, 14 with spots, but not certain ulceration, and 11 in which the presumptive evidence of ulceration was strong.

The average duration of theses cases, was—of the 26 cases, 10 days before admission and 21 days under treatment; of the 14 cases, 8 days before admission, and 22 under treatment; of the 11 cases, 7 days before admission, and 33 under treatment.

Turning now to those in which the quinia treatment was adopted, and almost invariably in ten grain doses every four hours, they include 5 cases in which there was pretty conclusive evidence of ulceration of the bowels, 3 of which were also spotted; 4 cases with spots, where ulceration was not proved, and only 2 in which neither condition was exhibited. With the last cases it may be best to classify the two already referred to under the second division, because they are not marked by any very broad line of distinction separating them from the present series, and they exhibit the quinia treatment under its most favorable view.

There are, therefore, 4 cases without spots or decided ulceration, of which the average duration was eight days before admission, and twenty-three under treatment; 4

cases with spots only, of which the average duration was ten days before admission, and twenty-six under treatment; 5 cases with ulcerated bowels, of which 3 had also spots, and their average is fifteen days before admission, thirty-seven under treatment.

I must here distinctly state, that when I commenced this report I had no idea what the result would be, and, so far from believing it unfavorable, had hoped that excluding some unfortunate cases, the treatment of fever with quinia would prove rather more speedy, safe, and effectual, than by the ordinary modes. I am sorry to be convinced that it has no advantages.

It may be well to state in conclusion, tnat the prevalent type of fever has been what would be called "typhoid," not true "typhus." One or two had the aspect of congestive typhus, but wanted the purple, mottled rash. One patient had this rash very well marked, mixed with ecchymosed spots, at the same time had distinct ulceration of the bowels, with a chapped and glazed tongue. Some had a very abundant crop of florid, slightly-elevated spots disappearing on pressure; some had only one or two of this character. Occasional y the spots are characterized as large, sometimes as small; and individual instances occur exhibiting various degress of persistency, and various sha^des of color, from a pale rose to a deep crimson. Spots existed without ulceration, and ulceration without spots, apparently without any definite rule; and some.of the most severe and tedious cases were unaccompanied by either one or the other.

4. [In commenting upon these marks, Dr. Dundas thinks] that, instead of contravening, they go far to substantiate the doctrine in question,—at least in so far as relates to the two only cases of fever in which "cinchonism' appears to have been *early* adopted. As regards the case with "ulcertion of the bowels," who expects, or who ever proposes, to *cure* such cases by cinchonism? I well know that cinchonism will not *arrest* such complications; but I equ lly well know that, resorted to in time, it will commonly prevent them.

Of three fatal cases in which cinchonism was resorted to (though I cannot clearly make out if they are included in Dr. Barclay's Analysis), one was "*apparent* typhus;" another "tubercular inflammation of the brain," and the third was "complicated with albuminuria," (nephritis?) Surely none of these cases can be adduced as affording any evidence on the present question.

It would seem that, in some of the cases, purgatives, had been administered; these, in my experience, prove almost invariably hurtful in fever. Were emetics given? The amount of support and stimulants,—another important consideration,—afforded to the patients under quinia does not appear.

Dr. Barclay admits the effect of cinchonism to be "something strange,"—that is, new to him; but, "in one or two cases which got well very speedily under its employment,"he questions" special effect:"—or, "if any," that such effect "was limited to lowering the general circulation;' that "when pushed to its full extent," it prostrates "the vital powers." On these points my experience is totally at variance with that of Dr. Barclay.

I have myself administered quinia to cinchonism in many hundred cases, and seen it largely given by others; yet in no one instance have I ever observed it cause "prostration of the vital powers," though often vomiting.

That the physiological effect of the remedy is not, as Dr. Barclay avows, "limited to lowering the general circulation," is proved by the fact, that commonly the general symptoms improve, not only *parri passu* with the change in the circulation, but that sometimes one, sometimes another, and occasionally *all* of the following changes will occur; namely, the tongue will become moist, and the thirst abated, the skin cooler, and the headache and general uneasiness diminished, *before* any decided change in the frequency or character of the pulse can be detected. Also, in the more favorable cases, the physiological effect of the remedy is displayed solely in the rapid and *simultaneous* subsidence of all the urgent symptoms; there will be no tinnitus aurium, no headache, no dizziness, no deafness, and no vomiting.

The perusal of Dr. Barclay's interesting paper, as well as what I constantly hear and witness, proves to me the necessity of once more placing briefly before the profession some of those points in the treatment of continued fever by cinchonism on which I have already urgently insisted —orally, and in my several publications,—during the last three years. As these points are in nothing modified by subsequent experience, I shall give the precise words in which I originally stated them, under the impression that this mode of recalling the subject may be more advantageous than any new or more lengthened statement :—

"I would here beg to recall, briefly, a few of those principles on which I have elsewhere strongly insisted, namely, that the value of cinchonism in typhus will be in proportion to its early induction ; that adopted early, it arrests with certainty, in the vast majority of cases, the course of all continued fevers, and thus presents the complications which prolong the disease and peril life ; that we cannot arrest all cases of typhus fever by cinchonism, nor can we all cases of ague; serious visceral disease, in either case, will interrupt the specific power of the remedy ; also idiosyncrasy in some, and a broken-down state of the constitution in others, will prevent its success; that a vital organ already seriously damaged, or the vital fluids already seriously vitiated, will necessarily render the success of cinchonism doubtful ; but that in none of the foregoing conditions, idosyncrasy excepted, should the remedy be altogether suspended. for even in these, its administration will prove commonly useful, and *always* safe ; that after the first impression has been made on the disease by cinchonism, the patient should be constantly and *well supported* :—no slops. Wine will be often necessary, and (especially with the hospital patients) brandy. To the purely medical measures I need not refer, but there is one point on which I am anxious to fix the attention of the profession, namely, that in estimating the specific power of cinchonism over typhus fever the practitioner *must carefully distinguish those cases of visceral disease, attended with low inflammation and typhoid symptoms*, which are continually admitted into hospitals as 'typhus,' or 'typhoid' fevers. In these cases the failure of cinchonism attaches, not to the remedy, but to the physician."

I may, perhaps, be permitted to add, that the dose I first adopted, namely, 10 or 12 grains every two hours to an adult, is that which I still find to be the most uniformly advantageous, and attended with the fewest inconveniences to the patient.—*Med. Times and Gaz. Jan.* 1853

